# Evaluation of a clinical pharmacist-led antimicrobial stewardship program in a neurosurgical intensive care unit: a pre-and post-intervention cohort study

**DOI:** 10.3389/fphar.2023.1263618

**Published:** 2023-09-22

**Authors:** Jing Yu, Yan Liu, Ruochen Qu, Ziyang Wang, Yan Zhao, Yuanyuan Zhao, Chunhua Zhou

**Affiliations:** ^1^ Department of Clinical Pharmacy, The First Hospital of Hebei Medical University, Shijiazhuang, China; ^2^ The Technology Innovation Center for Artificial Intelligence in Clinical Pharmacy of Hebei Province, The First Hospital of Hebei Medical University, Shijiazhuang, China; ^3^ Department of Clinical Pharmacy, Hebei Medical University, Shijiazhuang, China

**Keywords:** microbial, mortality, antimicrobial stewardship, neurosurgical intensive care units, drug resistance

## Abstract

**Background:** Antimicrobial resistance poses a significant challenge in neurosurgical intensive care units (ICU). The excessive use of broad-spectrum antibiotics is closely linked to the emergence and dissemination of drug-resistant bacteria within neurosurgical ICUs. This study assessed the effects of implementing a comprehensive Antimicrobial Stewardship (AMS) program in a neurosurgical ICU setting.

**Methods:** From April 2022 to September 2022, an AMS program was implemented in the neurosurgical ICU. The program involved the regular presence of a pharmacist and an infectious disease physician who conducted prospective audits and provided feedback. To assess the impact of the AMS program, the outcome measures were compared between the AMS period and the 6 months before AMS implementation (pre-AMS period). The primary outcome was the use of antibacterial agents, including anti-pseudomonal beta-lactams (APBLs), polymyxin, and tigecycline. Additionally, the study evaluated the appropriateness of antimicrobial de-escalation and the susceptibility of Gram-negative bacilli to antimicrobial agents.

**Results:** A total of 526 were included during the AMS period, while 487 patients were included in the pre-AMS period. The two groups had no significant differences in disease severity and mortality rates. During the AMS period, there was a notable decrease in the use of APBLs as empiric treatment (43.92% vs. 60.99%, *p* < 0.001). Multi-drug resistant organism (MDRO) infections decrease significantly during AMS period (11.03% vs. 18.48%, *p* < 0.001). The number of prescription adjustment increased significantly in all patients (0 item vs. 0 item, *p* < 0.001) and MDRO-positive patients (3 items vs. 2 items, *p* < 0.001) during the AMS period. Additionally, appropriate antimicrobial de-escalation for patients with MDRO showed improvement during the AMS period (39.66% vs. 20%, *p* = 0.001). Polymyxin utilization also decreased during the AMS period (15.52% vs. 31.11%, *p* = 0.034). Furthermore, the susceptibility of Gram-negative Bacilli isolates to APBLs was significantly higher during the AMS period.

**Conclusion:** Implementing a comprehensive pharmacist-led AMS program led to a decrease in the use of antibacterial agents. This reduction in usage is significant because it can potentially delay the emergence of bacterial resistance.

## 1 Introduction

Intensive care units (ICUs) are among the most common hospital areas where antibiotics are administered. They also play a crucial role in detecting and controlling multidrug-resistant organisms (MDROs), particularly bacteria (Versporten et al., 2018). Many antibiotics are administered improperly in ICUs, with estimates ranging from 30% to 60% (Trivedi et al., 2020). Epidemiological studies have consistently demonstrated a strong link between the irrational use of antibiotics and the emergence and spread of drug-resistant bacteria (Trivedi et al., 2020; National Health Commission of the People’s Republic of China, 2018). In light of these findings, implementing Antimicrobial Stewardship (AMS) Programs is highly recommended in ICUs. AMS aims to promote the rational use of antibiotics and improve patient outcomes (Barlam et al., 2016; Pickens and Wunderink, 2019). A well-executed AMS can have several positive therapeutic effects, including reducing antibacterial therapy duration, antibiotic consumption, hospital stay, healthcare costs, and the incidence of healthcare-associated infections (Ntagiopoulos et al., 2007; Feazel et al., 2014; Lee et al., 2019).

The neurosurgical ICU at our hospital is a semi-enclosed ICU with 22 beds, primarily serving patients who have undergone surgery in the neurosurgical department. Post-neurosurgical meningitis is showing an upward trend, and the increasing prevalence of MDRO affects outcomes following nosocomial meningitis (15van de Beek et al., 2010). Pulmonary infection is also a common complication after neurosurgery. According to relevant studies, the incidence of pneumonia with MDRO also exceeds 10% in patients with mechanical ventilation in the neurosurgical ICU (Teng et al., 2022). In 2021, the neurosurgical ICU had a markedly higher detection rate of MDRO compared to other ICUs within our hospital, with a rate of 18.48%. This alarming finding has prompted the hospital’s AMS team to take action. The occurrence of MDRO in the neurosurgical ICU is primarily attributed to bacterial variation and excessive use of antibiotics in clinical practice. To address this problem, our task force has proposed several critical interventions based on previous studies that assessed AMS’s effectiveness in acute care settings. These interventions include order review and feedback, multidisciplinary management, and whole-process monitoring. Although individual interventions have been studied in previous research, there is a lack of comprehensive assessments that evaluate the impact of a comprehensive AMS program (Lee et al., 2019), especially in the neurosurgical ICU setting.

Therefore, our study aimed to implement a comprehensive AMS program in the neurosurgical ICU and evaluate changes in antibiotic use, bacterial resistance patterns, and patient outcomes before and after implementing the program. By collecting and analyzing these data, we aimed to provide evidence that supports the effectiveness of AMS in the neurosurgical ICU setting and contributes to improving patient care.

## 2 Materials and methods

### 2.1 Study design

A pre-and post-intervention study was conducted at a tertiary academic hospital in China. The study spanned October 2021 to March 2022 (pre-AMS) and April 2022 to September 2022 (AMS). All patients admitted to the neurosurgical ICU for >24 h during the two study periods were included. The research protocol received approval from the Hospital Ethics Committee (approval number 20220115) on 27 January 2022。

### 2.2 The pre-AMS stage and the components of the AMS program

Before the implementation of AMS, patients were primarily managed by physicians without the supervision of the AMS team. In April 2022, a comprehensive pharmacist-led AMS program was introduced to the neurosurgical ICU. The AMS team included a clinical pharmacist (the program director), an infectious disease physician, an infection control specialist, a clinical microbiology laboratorian, and a nurse. This AMS team actively engaged in the daily management of patients, with particular attention given to those suffering from multi-drug resistant infections. The suggestions proposed by the AMS team were thoroughly discussed and agreed upon by the attending physicians, ensuring a collaborative approach to patient care.

Clinical pharmacists held a discussion meeting prior to the start of AMS program to outline the duties of members and the specific implementation strategy (Table 1). Aspiration pneumonia is the most common infection in neurosurgical ICU and is mostly community-acquired. Physicians in neurosurgical ICU used piperacillin/tazobactam as an empiric treatment. The AMS team decide to use amoxicillin/clavulanate as empirical treatment based on the bacteria found in earlier respiratory specimens and their resistance.

**TABLE 1:** The content of the antimicrobial stewardship (AMS) program in the neurosurgical ICU.

Project	Time	Measure	
Patient management	Patients transferred to neurosurgical ICU	1. The AMS team develops antimicrobial treatment plans based on applicable guidelines and the specific circumstances of the patients. These plans are tailored to optimize the use of antimicrobial agents while considering factors such as the type of infection, pathogen susceptibility patterns, and individual patient characteristics	
		2. Procalcitonin (PCT) levels in the patients’ serum are assessed on the third and seventh day after surgery. If the PCT level is below 0.5 ng/mL or has decreased by more than 80% compared to previous measurements, antibiotic administration is discontinued. This approach helps guide the appropriate duration of antibiotic therapy and prevent unnecessary exposure to antimicrobial agents	
		Medical advice review and communication	Clinical pharmacists; daily
		Ward rounds	AMS team; every 3 days
	MDRO-detected patients	1. Adherence to quarantine requirements	
		2. Assessment of bacterial infection or colonization: by accurately identifying and differentiating between infections and colonization, healthcare providers can make informed decisions regarding antimicrobial therap	
		3. Monitoring and adjustment of treatment: regular monitoring of key indicators such as blood counts, total biochemical values, PCT, and signs of infection is essential. These measurements are typically conducted every 3 days after infection. Based on the patient’s clinical signs and infection status, the AMS team makes the necessary adjustments to the administration regimen, ensuring optimal treatment outcomes	
		Full process monitoring	Clinical pharmacists played a crucial role in patient care by monitoring various aspects of medication management, including the implementation of medication plans, efficacy monitoring; patient adherence monitoring; genetic testing and interpretation; and therapeutic drug monitoring
		Ward round	AMS team; 1–2 times daily
Infection prevention	1. Nosocomial infection experts monitor and ensure the effective implementation of infection prevention and control procedures in neurosurgical ICU.		
	2. Physicians and nurses receive instructions from clinical microbiology laboratories on proper collection of culture specimens in a standardized manner. It aimed to ensure the accurate and reliable identification of pathogens causing infections in neurosurgical ICU patients		
Monitor ADRs	Nurses are assigned the responsibility of monitoring and promptly reporting any adverse events that occurred in patients		
Regular meetings	1. Once every 2 weeks		
	2. Outcome measures closely monitored and evaluated. These measures assess the impact of AMS on various aspects of antibiotic use, physician prescribing practices, MDRO detection, patient outcomes, and adverse events		
	3. Discussion of typical cases		
	4. Identify areas of improvement in the next phase of the plan		

AMS: antimicrobial stewardship; MDRO: Multi-drug resistant organism; ICU: intensive care unit; ADRs: Adverse drug reactions.

During the AMS period, clinical pharmacists performed pharmaceutical care activities as well as organizing, supervising, and recording. Every 3 days, pharmacists and physicians conducted ward visits for all patients in neurosurgical ICU. For MDRO-positive patients, ward rounds were performed 1–2 times a day. Physicians moderated the rounds and developed or modified the antimicrobial use with pharmacists. Clinical pharmacists played a crucial role in patient care by monitoring various aspects of medication management, including the implementation of medication plans, efficacy monitoring, patient adherence monitoring, organizing multidisciplinary consultations (MDT) for critically ill patients and supervising the implementation of consultation opinions. Additionly, the clinical pharmacy laboratory in hospital had carried out targeted sequencing detection (tNGS) and therapeutic drug monitoring (TDM) of antibiotics. Clinical pharmacists are responsible for determining the timing of examination monitoring and the interpretation of relevant reports.

The execution of infection prevention and control measures in the neurosurgical ICU was supervised and ensured by experts in nosocomial infections. The microbiologist provided guidance on the proper collection of specimens. The nurse was in charge of monitor and promptly reporting any adverse events that occurred in patients.

AMS meeting was held every 2 weeks, to share data of the recent MDRO detection rate, the application of antibacterial drugs and ADRs. Typical cases, practical problems and the goals for the next 2 weeks were also discussed.

### 2.3 Data collection

Demographic and clinical data were collected through medical record reviews and patient interviews. The following data were collected: age, sex, length of stay, comorbidities, site of infection (presumed or confirmed), disease severity, white blood cell count, procalcitonin, alanine transaminase, aspartate transaminase, serum creatinine, bacterial organisms, adverse drug reactions, and antibiotic information.

### 2.4 Primary outcomes

The primary outcome was the use of *β*-lactam drugs targeting *Pseudomonas Aeruginosa*, including ceftazidime, piperacillin/tazobactam, cefoperazone/sulbactam, cefepime, imipenem-cilastatin, or meropenem. This was assessed by calculating the total antibiotic days of therapy (DOT) per 1,000 patient days, represented as DOT/1,000 patient days (Devlin and Mckenzie, 2018). DOT is determined in two steps. First, the number of doses that the patient receives is multiplied by the dosing interval (Lu et al., 2019). Second, this value is divided by 24 h to calculate the DOT for each antibiotic received by the patient (Lu et al., 2019; Hamdy et al., 2020). For patients who tested positive for MDRO, the proportion of individuals receiving polymyxin and tigecycline antibiotics was determined, together with the number of days of treatment for each patient.

### 2.5 Secondary outcomes

Secondary outcomes were hospital mortality, all-cause readmissions, readmissions due to the exact cause of the previous admissions, antibiotic prescription adjustment, appropriate de-escalation, and adverse drug reactions during the study period.

### 2.6 Statistical analyses

Data processing and analyses were performed using Microsoft Excel 2019 (Microsoft Corp., Redmond, United States) and SPSS 26.0 statistical software (IBM Corp., Chicago, United States). Normally distributed measurement data are expressed as means ± standard deviations (SD), and independent-sample t-tests were used for group comparisons. If the data involved paired samples, a paired sample *t*-test was conducted. Non-normally distributed measurement data are presented as medians with interquartile ranges (IQR), and the Mann-Whitney *U* test was used for group comparisons. The Wilcoxon paired rank sum test was employed for paired samples with non-normal distribution. Categorical and ranked data are described by numbers and percentages. The chi-square test was used for group comparisons, while the Wilcoxon rank sum test was applied for ranked data.

## 3 Results

### 3.1 Study population

A total of 1,013 patients were admitted to the neurosurgical ICU for more than 24 h, 487 in the pre-AMS group and 526 in the AMS group. Table 2 shows the baseline characteristics of the patients in each group. The AMS group had a significantly higher proportion of patients with hypoproteinemia than the pre-AMS group (38.02% vs. 14.99%, *p* < 0.001). Furthermore, the AMS group had a higher incidence of urinary infections than the pre-AMS group (4.94% vs. 1.85%, *p* = 0.009).

**TABLE 2:** Baseline characteristics of the study population.

	AMS (*n* = 526)	Pre-AMS (*n* = 487)	*p*-value
Sex (male), n (%)	347 (65.97)	297 (60.99)	0.103
Age (mean, IQR), years	59 (50–69)	59 (49–69)	0.593
ICU stay (mean, IQR), days	5 (3–10)	7 (4–10)	0.126
Surgery before ICU admission, n (%)	441 (83.84)	399 (81.93)	0.452
Underlying disease, n (%)			
Diabetes mellitus	68 (12.93)	63 (12.94)	1.00
Metastatic cancer	10 (1.90)	9 (1.85)	1.00
Liver cirrhosis	31 (5.89)	34 (6.98)	0.522
Chronic kidney disease	36 (6.84)	24 (4.93)	0.231
Hypoproteinemia	200 (38.02)	73 (14.99)	<0.001
Infection site (presumed or confirmed), n (%)			
Superficial surgical site	1 (1.90)	4 (0.82)	0.201
Respiratory	383 (72.81)	379 (77.82)	0.069
Urinary tract	26 (4.94)	9 (1.85)	<0.001
Catheter-related	2 (0.38)	4 (0.82)	0.436
Intracranial	21 (3.99)	19 (3.90)	1.00
Disease severity			
Sepsis	11 (1.52)	9 (0.82)	0.825
Septic shock	8 (1.52)	6 (1.23)	0.791
APACHE Ⅱ, mean (SD)	17.05 ± 12.00	16.49 ± 11.95	0.462

AMS: antimicrobial stewardship; ICU: intensive care unit; IQR: interquartile range; APACHE Ⅱ: Acute physiology and chronic health evaluation Ⅱ.

A total of 148 patients were tested positive for MDRO, 90 in the pre-AMS group and 58 in the AMS group. The prevalence of MDRO-positive patients was significantly higher in the pre-AMS group compared to the AMS group (18.48% vs. 11.03%, *p* = 0.001). These MDRO-positive patients were analyzed separately. Table 3 provides an overview of the baseline characteristics of the patients with MDRO. Among MDRO-positive patients, more patients in the AMS group had diabetes mellitus (24.10% vs. 10.00%, *p* = 0.035) and septic shock (6.9% vs. 0%, *p* = 0.022) compared to the pre-AMS group. The proportion of patients with MDRO *Klebsiella pneumoniae* was lower in the AMS group (63.79% vs. 81.11%, *p* = 0.022).

**TABLE 3:** Baseline characteristics of patients with positive multi-drug resistant organisms.

	AMS	Pre-AMS	*p*-value
MDRO infections	58 (11.03)	90 (18.48)	<0.001
Sex (male), n (%)	23 (36.7)	38 (42.2)	0.864
Age (mean, IQR), years	62.5 (53.75–72)	61.5 (50–71)	0.560
ICU stay (mean, IQR), days	11 (9–17)	11.5 (7–16)	0.780
Underlying disease, n (%)			
Diabetes mellitus	14 (24.1)	9 (10.0)	0.035
Metastatic cancer	0 (0)	1 (1.1)	1
Liver cirrhosis	3 (5.17)	5 (5.56)	1
Chronic kidney disease	6 (10.3)	5 (5.6)	0.341
Hypoproteinemia	35 (60.3)	49 (84.5)	0.501
MDRO, n (%)			
*Acinetobacter baumannii*	11 (18.97)	23 (25.56)	0.425
*Klebsiella pneumoniae*	37 (63.79)	73 (81.11)	0.022
*Pseudomonas aeruginosa*	6 (10.34)	9 (10.00)	1
*MRSA*	8 (13.8)	8 (8.9)	0.421
Others	5 (8.62)	1 (1.11)	0.034
Infection site (presumed or confirmed)			
Respiratory	54 (93.1)	78 (86.7)	0.283
Urinary tract	4 (6.9)	9 (10.0)	0.569
Intracranial	0 (0)	6 (6.67)	0.082
Disease severity			
Sepsis	8 (13.8)	4 (4.4)	0.062
Septic shock	4 (6.9)	0 (0)	0.022
APACHE Ⅱ, mean (SD)	24.60 ± 7.71	22.71 ± 5.41	0.081

MDRO: Multi-drug resistant organism; MRSA: Methicillin-resistant *Staphylococcus aureus*.

### 3.2 Assessment of the primary outcome

**FIGURE 1:**
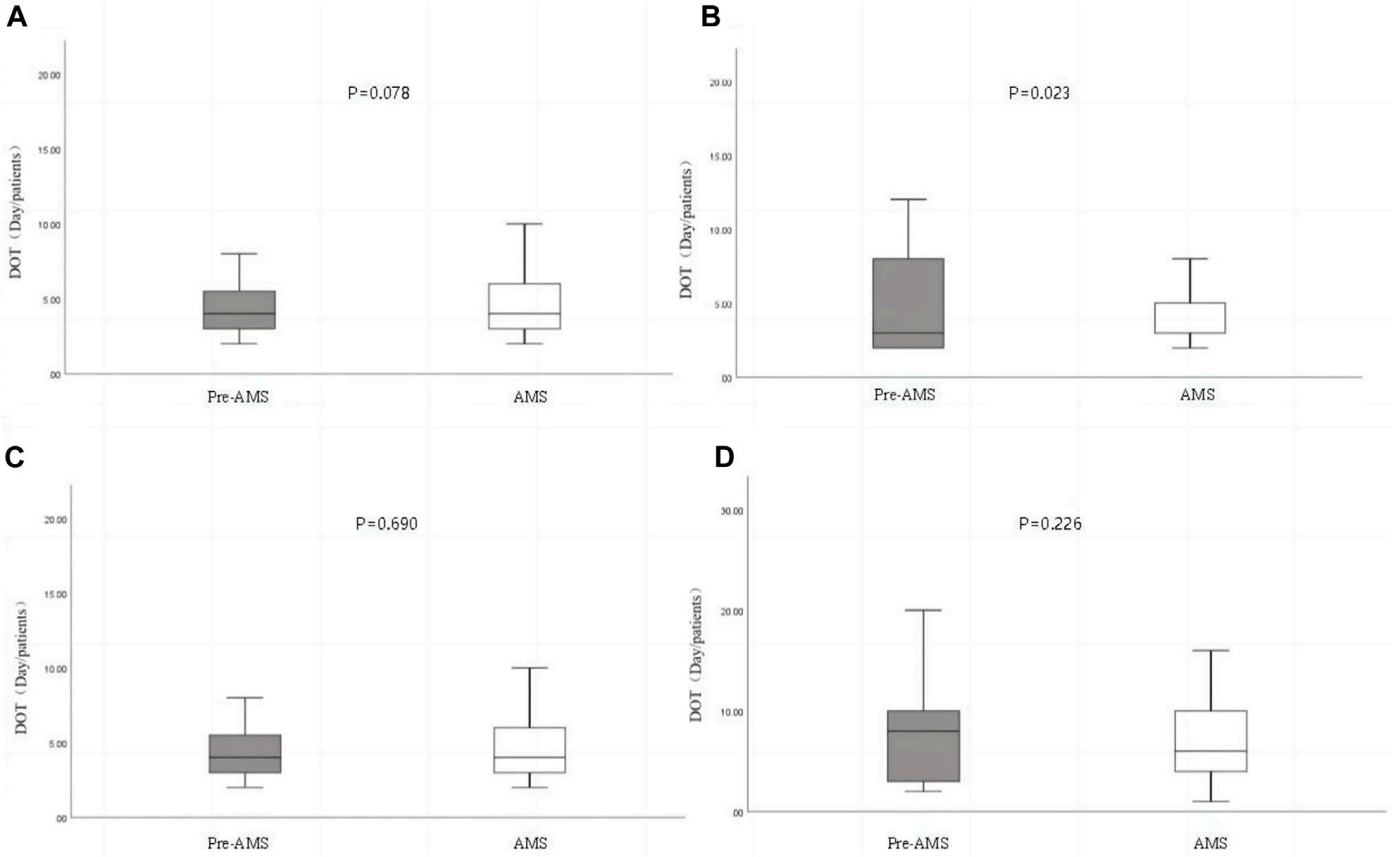
Days of therapy (DOT) of anti-pseudomonal beta-lactams (APBL) during the pre-antimicrobial stewardship program (AMS) and AMS periods. The cefepime data are removed due to the lack of cefepime from September to November 2021. **(A)**. Ceftazidime; **(B)**. cefoperazone-sulbactam; **(C)**. piperacillin/tazobactam; **(D)**. meropenem + imipenem.

Fewer patients received empiric anti-pseudomonal beta-lactam (APBL) treatment in the AMS group compared to the pre-AMS group (43.92% vs. 60.99%, *p* < 0.001). However, no significant differences were observed in the proportion of APBL use as a definitive treatment (42.97% vs. 40.86%, *p* = 0.524) and DOT (8.85 days vs. 8.7 days, *p* = 0.780) of APBL between the AMS and pre-AMS groups. Details are shown in Table 4. When assessing the unit-wide use of APBL, the DOT of cefoperazone/sulbactam was significantly lower in the AMS group (4.64 days vs. 5.54 days,*p* = 0.023, Figure 1). The proportion of MDRO-positive patients who received polymyxin treatment was significantly lower in the AMS group (15.52% vs. 31.11%, *p* = 0.034, Table 5).

**TABLE 4:** Comparison of clinical outcomes of patients admitted to the neurosurgical intensive care unit during the pre-antimicrobial stewardship (AMS) program and AMS periods.

	AMS (*n* = 526)	Pre-AMS (*n* = 487)	*p*-value
Primary outcomes			
Use of anti-pseudomonal beta-lactams as empirical treatment (≥2 days), n (%)	231 (43.92)	297 (60.99)	<0.001
Use of anti-pseudomonal beta-lactams as definitive treatment (≥2 days), n (%)	226 (42.97)	199 (40.86)	0.524
DOT of anti-pseudomonal beta-lactams (day/patients), mean (SD)	8.85 ± 7.10	8.7 ± 6.43	0.780
Secondary outcomes, n (%)			
Mortality	55 (10.46)	60 (12.32)	0.373
All-cause readmissions	28 (5.32)	12 (2.46)	0.023
Readmissions due to the same cause as the previous admission	13 (2.47)	9 (1.85)	0.526
Prescription adjustment patients	245 (46.58)	198 (40.66)	0.058
Prescription adjustment number (mean, IQR),items	0 (0,2)	0 (0,1)	<0.001
Appropriate de-escalation	147 (27.95)	112 (22.30)	0.072
Adverse drug reactions	60 (11.41)	63 (12.94)	0.501

AMS: antimicrobial stewardship; DOT: Days of therapy.

**TABLE 5:** Comparison of clinical outcomes of patients with positive multi-drug resistant organism.

	AMS (*n* = 58)	Pre-AMS (*n* = 90)	*p*-value
Primary outcomes			
Use of Polymyxin (≥2 days), n (%)	9 (15.52)	28 (31.11)	0.034
DOT of Polymyxin (day/patients), mean (SD)	11.00 ± 5.07	13.18 ± 7.64	0.431
Use of Tigecycline (≥2 days), n (%)	2 (3.45)	10 (11.11)	0.127
DOT of Tigecycline (day/patients), mean (SD)	5.00 ± 2.83	8.50 ± 5.91	0.445
Secondary outcomes, n (%)			
Mortality	13 (22.41)	24 (26.67)	0.698
All-cause readmission	7 (12.07)	4 (4.4)	0.111
Readmission due to the same cause as the previous admission	2 (3.45)	0 (0)	0.152
Prescription adjustment number (mean, IQR),items	3 (3,4)	2 (2,3)	<0.001
Appropriate de-escalation	23 (39.66)	18 (20.00)	<0.001
Adverse drug reactions	7 (12.07)	18 (20%)	0.264

### 3.3 Mortality and other secondary outcome measures

There were no significant differences in mortality between the AMS and pre-AMS groups (10.46% vs. 12.32%, *p* = 0.373). Regarding neurosurgical ICU readmissions, the AMS group had a higher frequency than the pre-AMS group (5.32% vs. 2.46%, *p* = 0.023). However, when considering readmissions with an exact cause as previous admission, there was no statistically significant difference between the AMS and pre-AMS groups (2.47% vs. 1.85%, *p* = 0.526). Details are shown in Table 4.

**FIGURE 2:**
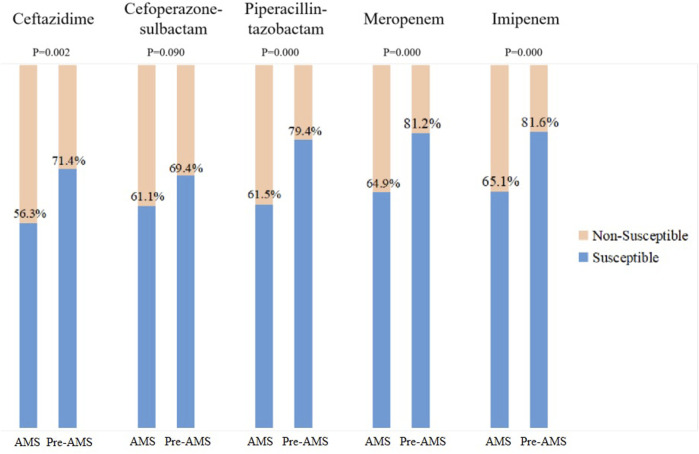
Anti-pseudomonal beta-lactam susceptibility of Gram-negative Bacilli during pre-antimicrobial stewardship program (AMS) and AMS periods. Overall antimicrobial susceptibility of five common Gram-negative bacilli (*Escherichia coli*, *Klebsiella pneumoniae*, *Enterobacter cloacae* complex, *Pseudomonas aeruginosa*, and *Acinetobacter baumannii*).

There was no significant difference in the proportion of patients with prescription adjustment. But the number of prescription adjustment increased significantly in all patients (0 item vs. 0 item, *p* < 0.001) and MDRO-positive patients (3 items vs. 2 items, *p* < 0.001) during AMS period. Appropriate antimicrobial de-escalation did not improve significantly during the AMS period since the initial medication has been changed in the AMS group (27.95% vs. 22.3%, *p* = 0.072). However, when focusing on MDRO-positive patients, there was a significant improvement in the appropriate antimicrobial de-escalation during the AMS period compared to the pre-AMS period (39.66% vs. 20.00%, *p* = 0.001, Table 5). Furthermore susceptibility of GNB(Gram-Negative Bacilli) isolates to APBLs in AMS period was significantly higher, except cefoperazone-sulbactam (Ceftazidime: 56.3% vs. 71.40%, *p* = 0.002; Piperacillin/tazobatam: 61.5% vs. 79.40%, *p* <0.001; Meropenem: 64.90% vs. 81.20%, *p* <0.001; Imipenem: 65.10% vs. 81.60%, *p* <0.001; Figure 2).

## 4 Discussion

The neurosurgical ICU accepts only post-neurosurgical patients. Most of these patients had significant intraoperative blood loss, received blood transfusions, and remained unconscious for a long time after operation (Sachdeva et al., 2017). These conditions are independent risk factors for pulmonary infection. Meanwhile, meningitis is one of the main complications following neurosurgery (Li et al., 2016; Iwasaki et al., 2017). According to reports, the incidence ranges from 0.3% to 6.6% (Chen et al., 2014; Ma et al., 2019) and is particularly high in developing countries. Even the incidence of catheter-associated urinary tract infections in the neurosurgical ICU was the third highest among all ICUs, reaching 5.28% (Ma et al., 2019). All of them result in prolonged hospital stays and increased antibiotic usage, which eventually give rise to MDRO. As with all neurosurgical ICUs, our neurosurgical ICU is staffed not only by intensivists, but also by neurosurgeons, who are not good at postoperative management and using antibiotics. Therefore, it is necessary to implemente a comprehensive AMS program in the neurosurgical ICU and there are few related studies.

This study examined the impact of a comprehensive AMS program led by clinical pharmacists on antibiotic utilization, bacterial resistance, and patient outcomes in a neurosurgical ICU. Implementing this comprehensive AMS program demonstrated significant benefits, including reducing broad-spectrum antibiotics (APBLs) use within the neurosurgical ICU setting. Furthermore, it led to a decrease in the detection rate of multi-drug-resistant bacteria.

The use of antibiotics in ICUs is considerable, and studies indicate that up to 70% of ICU patients receive antibiotics (Gruenberg et al., 2006; Park et al., 2017). The average consumption of antibiotics per 1,000 days of hospital stays in ICUs is reported to be 1,563 defined daily doses, three times higher than in normal wards (Bitter et al., 2016). Alarmingly, more than 50% of antibiotics administered in the ICU are deemed inappropriate (Rhodes et al., 2017). Furthermore, over one-third of non-infected patients receive unnecessary antibiotics (Lam et al., 2018; van Someren et al., 2018). Implementing an AMS program has demonstrated the potential to reduce antimicrobial usage in ICUs without increasing mortality rates (Lindsay et al., 2019). These findings are consistent with the conclusions drawn from our study, which revealed a significant decrease in the proportion of empiric treatment with broad-spectrum antibiotics (APBLs) in patients while maintaining a similar mortality rate before and after AMS implementation, despite the difference of the patient severity. Early and aggressive intervention seemed to contribute to disease severity of patients during the AMS period.

The emergence and spread of antimicrobial resistance can be attributed to various factors. However, antibiotic usage is essential in bacterial resistance, particularly when highly potent antibacterial agents with broad-spectrum activity are used over extended periods. Research has consistently demonstrated a correlation between increased piperacillin/tazobactam use and an 8% increased risk of multi-drug-resistant bacteria (Teshome et al., 2019). Furthermore, the use of imipenem has been associated with the development of substantial resistance to bacterial drugs (Armand-Lefèvre et al., 2013). Our study underscores the importance of promoting appropriate de-escalation practices, which can significantly reduce the detection of MDROs and the resistance rate of common clinically relevant Gram-negative bacteria such as *Klebsiella pneumoniae, Escherichia coli, Acinetobacter baumannii, Pseudomonas aeruginosa, and Enterobacter cloacae*, against antimicrobial agents. By effectively administering antibiotics and evaluating their efficacy, we can effectively delay the progression of bacterial resistance. And this improvement continued, half a year after the end of the study, the detection rate of MDRO in neurosurgical ICU was 14.71%. Due to the outbreak of COVID-19 infection in China from January to March 2023, MDRO infections increased. We also collected the detection rate of MDRO in neurosurgical ICU from April to July 2023 was 11.11%, indicating that the detection rate of MDRO had decreased significantly and remained stable. During the same period, GNB maintained a high sensitivity to APBLs (Ceftazidime: 67.57%; Piperacillin/tazobatam: 85.56%; cefoperazone/sulbactam: 77.48%; Meropenem: 89.19%; Imipenem: 89.19%).

Implementing an AMS program in ICUs poses specific challenges. Clinical pharmacists play a crucial role as integral members of the AMS team, and in our case, the AMS director is a clinical pharmacist. Their expertise in pharmacokinetics and pharmacodynamics is essential to accurately adjust antibiotic dosages in ICU patients. Factors such as molecular weight and fat-soluble protein binding properties of drugs influence their distribution within the body and at the site of infection. Individualized administration of antibacterial agents must consider the patient’s organ function, disease severity, source of infection, and other relevant factors (Chua et al., 2022). A study by MacLaren et al. revealed that a shortage of clinical pharmacists in the ICU contributes to increased mortality among infected patients (Maclaren et al., 2008). This emphasizes the importance of having adequate clinical pharmacists available in ICU settings. In our study, pharmacists assisted physicians in making more prescribing adjustments. It indicates that pharmacists can timely assist physicians to adjust individualized drug administration regimen according to the efficacy of anti-infection treatment, tNGS results and TDM results.

Our neurosurgical ICU exhibited relatively lower mortality rates in both groups compared to some previous studys (Lindsay et al., 2019). It is important to note that the neurosurgical ICU exclusively admits patients undergoing neurosurgery. Unlike general ICUs, sepsis, identified as one of the leading causes of mortality (Kashiouris et al., 2019), is rare in the neurosurgical ICU. Therefore, the lower mortality rates observed in our ICU can be attributed to the different disease profiles between the two settings. However, it should be noted that the neurosurgical ICU experiences an increased proportion of patients with intracranial infections, leading to higher overall use of antibacterial agents, as reflected in the DOT metric. Intracranial infection patients typically require a longer antibacterial treatment than those with other infections (Tamma et al., 2021).

Implementing a comprehensive AMS program with limited resources can be a burden. In this study, team members, including clinical pharmacists, dedicated much time to completing the necessary tasks. Clinical pharmacists had to allocate half a day to monitor patients in the neurosurgical ICU, and the time commitment was even more significant when regular meetings were scheduled. A Japanese study has suggested that a once-weekly intervention can effectively reduce the use of carbapenem antibiotics in the ICU (Hagiwara et al., 2018). Therefore, more research is required to assess the optimal frequency of implementing different interventions within AMS, achieving a balance between improving antimicrobial usage and minimizing the workload of medical staff. Furthermore, we only collected information on antimicrobial resistance after AMS to assess the persistence of the AMS program. More data of antimicrobial application should be collected to assess the sustainability of the AMS program.

## 5 Conclusion

Implementing a comprehensive pharmacist-led AMS program in a neurosurgical ICU yielded positive outcomes. The program successfully reduced the use of broad-spectrum antibiotics (APBLs) and increased the susceptibility of Gram-negative bacteria to antimicrobial agents. This achievement highlights the effectiveness of a collaborative approach involving a multidisciplinary team in promoting prudent antimicrobial use and improving patient outcomes within the neurosurgical ICU setting.

## Data Availability

The raw data supporting the conclusion of this article will be made available by the authors, without undue reservation.
